# Acetaminophen and warfarin: A recipe for supratherapeutic international normalized ratio with postoperative bleeding risk

**DOI:** 10.1016/j.jdcr.2023.10.028

**Published:** 2023-11-25

**Authors:** Justin Thrush, Robert Simonds, Ashley Antony, Allison Vidimos, Jennifer Lucas

**Affiliations:** Department of Dermatology, Cleveland Clinic, Cleveland, Ohio

**Keywords:** acetaminophen, analgesia, bleeding risk, complications, dermatologic surgery, dermatology, INR, postoperative management, warfarin

## Introduction

Many patients encountered in dermatologic surgery take warfarin for anticoagulation due to cardiovascular comorbidities. Postoperative analgesia is a common concern for both the patient and dermatologic surgeon. Acetaminophen is a commonly used perioperative analgesic in dermatologic surgery. When acetaminophen is combined with warfarin, supratherapeutic international normalized ratio (INR) may result in an increased risk of postoperative bleeding. This little-known interaction is reported in the pharmacology literature, but has yet to be reported in dermatologic surgery literature. Providers need to be aware that warfarin for anticoagulation and acetaminophen for analgesia, when taken together, present a risk of bleeding in dermatologic surgery.

## Discussion

Two patients treated with Mohs micrographic surgery (MMS) for nonmelanoma skin cancer at our institution were on warfarin for anticoagulation and took acetaminophen for pain control, without other drug or dietary changes, and subsequently developed supratherapeutic INR. Both patients required warfarin dose adjustment that affected their surgical course, both preoperatively and postoperatively.

Our first patient presented for MMS of a basal cell carcinoma on the right upper cutaneous lip. She took warfarin for anticoagulation given her history of antiphospholipid antibody syndrome, deep vein thrombosis, pulmonary embolism, and atrial fibrillation with an INR goal of 2.5 to 4.0. Her preoperative INR was 2.4 the day before surgery. After surgery, she took 1000 mg acetaminophen every 6 hours for pain control. Three days later, she presented with pain, swelling, and bleeding from her surgical site. Hemostasis was obtained with cautery and a pressure dressing due to continued bleeding. Given her postoperative bleeding and the difficulty in achieving hemostasis, it was recommended she have her INR checked. Her INR was 5.1, and warfarin was subsequently held until INR normalized to goal range. In the next 2 days respectively, her INR improved to 4.2, then 2.7, which was within goal range. Postoperative pain control was difficult for the patient. She did not tolerate opioid medications and was also unable to take nonsteroidal anti-inflammatory drugs. As a result, the patient ultimately returned for a right infraorbital nerve block with 1% lidocaine with epinephrine and 0.5% bupivacaine, which improved her pain.

The second patient was referred to dermatologic surgery for treatment of a basal cell carcinoma on the right side of the temple. She had a history of atrial fibrillation and was also on warfarin for anticoagulation with a stable INR of 2.0 to 3.0 for many months. She had recently been taking acetaminophen 1300 mg every 8 hours for a total of 3900 mg daily for back pain in the days before surgery. There were no other changes in her diet or medications. In accordance with the surgeons’ preoperative protocol requiring an INR within 1 week of surgery, 1 day before surgery, her INR was noted to be elevated at 7.2. Her surgery was subsequently postponed, warfarin was held, and acetaminophen discontinued until normalization of INR to her goal range of 2.0 to 3.0. Her INR remained within therapeutic goal range after discontinuation of acetaminophen, and she subsequently had successful MMS 1 month later. Tramadol 50 mg every 6 hours as needed was used as an alternative to acetaminophen for postoperative pain control given her prior INR elevations while taking acetaminophen and warfarin concurrently. The patient had no postoperative complications.

## Discussion

Acetaminophen taken concurrently with warfarin has been reported in the pharmacology literature to cause elevated INR, although reports are rare. A case-control study found acetaminophen ingestion to be independently associated in a dose-dependent manner with having an INR >6.0, therefore acetaminophen should be considered as a potential culprit for INR variability in patients taking warfarin.^1^

Various mechanisms by which concurrent use of acetaminophen leads to supratherapeutic INR in patients taking warfarin have been reported. One proposed mechanism is that acetaminophen metabolism indirectly leads to decreased warfarin metabolism by enzymatic shunting and competition for cytochrome P450 (CYP) isoforms, including CYP2E1, CYP1A2, and CYP3A4.^2^ It has also been proposed that ingestion of acetaminophen reduces the concentration of vitamin K dependent clotting factors and leads to elevated INR.^3^

Acetaminophen is the most utilized postoperative analgesic in dermatologic surgery.^4^ Common first-line regimens include 1 g acetaminophen by mouth every 6 hours (not to exceed 4 g in 24 hours) and 400 to 600 mg ibuprofen by mouth every 6 hours.^4^ Caution must be undertaken in patients who are at increased risk of bleeding, including patients on warfarin for anticoagulation. Alternatives to acetaminophen and nonsteroidal antiinflammatory drugs include opioid medications and procedural interventions such as nerve blocks. If opioids are prescribed, the lowest amount for the shortest duration to achieve adequate analgesia is recommended. A recent review suggested prescribing no more than 36 hours of opioid analgesia postoperatively, starting with 50 mg tramadol every 6 hours as needed.^4^ If refills beyond a 36-hour supply are requested, a return visit should be scheduled to evaluate the surgical site for other causes of prolonged pain. Should patients be unable to tolerate any of these options due to side effects, postoperative regional nerve blocks can be considered, as was the case in our first patient. The authors have also noted successful postoperative pain control with the addition of the longer acting anesthetic bupivacaine at the time of surgery. Bupivacaine use immediately following MMS has been shown to reduce postoperative pain and narcotic analgesic use.^5^

In summary, dermatologic surgeons should be aware of the potential interaction of acetaminophen and warfarin leading to supratherapeutic INR. In our cases, we believe the addition and duration of acetaminophen use concurrently with warfarin led to supratherapeutic INR levels via the aforementioned mechanisms. Opioid pain medications can be considered as an alternative, but should be limited to the lowest dose and shortest duration possible, given their potential for abuse and dependency. Postoperative regional nerve blocks, longer acting anesthetics, as well as ice and cool compresses can be considered if oral analgesic options are not tolerated. If there are concerns for postoperative bleeding after dermatologic surgery, close follow-up should be arranged with the surgeon and the provider managing the patient’s warfarin, as warfarin dosing may need to be adjusted and INR closely monitored.

## Conflicts of interest

None disclosed.
